# A Wearable System for Real-Time Continuous Monitoring of Physical Activity

**DOI:** 10.1155/2018/1878354

**Published:** 2018-03-20

**Authors:** Fabrizio Taffoni, Diego Rivera, Angelica La Camera, Andrea Nicolò, Juan Ramón Velasco, Carlo Massaroni

**Affiliations:** ^1^Unit of Biomedical Robotics and Biomicrosystems, Department of Engineering, Università Campus Bio-Medico di Roma, Via Álvaro del Portillo 21, 00128 Rome, Italy; ^2^Departamento de Automática, Escuela Politécnica Superior, Universidad de Alcalá, Campus Universitario, 28871 Alcalá de Henares, Madrid, Spain; ^3^Department of Movement, Human and Health Sciences, University of Rome “Foro Italico”, Piazza Lauro De Bosis 6, 00135 Rome, Italy; ^4^Unit of Measurements and Biomedical Instrumentation, Department of Engineering, Università Campus Bio-Medico di Roma, Via Álvaro del Portillo 21, 00128 Rome, Italy

## Abstract

Over the last decades, wearable systems have gained interest for monitoring of physiological variables, promoting health, and improving exercise adherence in different populations ranging from elite athletes to patients. In this paper, we present a wearable system for the continuous real-time monitoring of respiratory frequency (*f*_R_), heart rate (HR), and movement cadence during physical activity. The system has been experimentally tested in the laboratory (by simulating the breathing pattern with a mechanical ventilator) and by collecting data from one healthy volunteer. Results show the feasibility of the proposed device for real-time continuous monitoring of *f*_R_, HR, and movement cadence both in resting condition and during activity. Finally, different synchronization techniques have been investigated to enable simultaneous data collection from different wearable modules.

## 1. Introduction

Monitoring training is essential to optimizing performance, promoting health, and improving exercise adherence in different populations ranging from elite athletes to patients. Despite the widespread diffusion of wearable devices providing information on mechanical and physiological parameters related to training, technological development is not always supportive of training monitoring [1].

Coaches and practitioners potentially have a myriad of variables and training metrics to deal with, often recorded with different devices that are not synchronized, thus increasing the time needed to gain relevant information from data. This scenario results in a limited use of the available devices and facilities, which are often substituted by more straightforward solutions. An example of such decision-making process is the widespread choice of measuring perceived exertion using a subjective scale [1]. Perceived exertion limits endurance performance [2], affects exercise adherence in sedentary individuals [3], and is widely monitored during training [1]. Notwithstanding its importance, subjective rating of perceived exertion (RPE) should be used in combination with objective variables to allow for a more comprehensive description of the training process. Indeed, physiological and mechanical variables, unlike RPE, can be measured continuously during exercise and can also be real-time displayed.

Emerging evidence suggests that respiratory frequency (*f*_R_) is a very promising variable to monitor during exercise. *f*_R_ is a better marker of physical effort compared to traditionally monitored physiological variables (including HR) and is strongly correlated with RPE during both continuous exercise and high-intensity interval training [4–7]. Among a myriad of potential applications, monitoring *f*_R_ during training would, for instance, benefit those patients who have their HR altered by pharmacological interventions (e.g., beta blockers). While *f*_R_ is a better marker of physical effort than HR is, the latter is, unlike *f*_R_, strongly linked with oxygen uptake and thus provides information on aerobic contribution and energy expenditure. A possible solution combines different variables [8] providing information on physical effort, energy expenditure, and task-related mechanical variables (e.g., acceleration, speed, cadence), possibly using low-cost ubiquitous-spread technologies.

During the last decade, the use of sensors for monitoring human behavior has exponentially increased, and several familiar objects of daily life have become hidden measurement tools [9]. This trend has been fostered by the increased level of miniaturization in sensor-manufacturing processes from the one hand and by the increasing computational availability on the other hand. In particular, the widespread distribution of tiny, yet relatively powerful computers, such as those embedded in smartphones, has enabled ubiquitous data acquisition and analysis [10]. Currently available smartphones and laptops are provided with different machine-to-machine (M2M) interfaces that have enabled the possibility to acquire synchronous heterogeneous data from several sensors in a relatively small space range, like in fitness training.

The aim of this work is to design, develop, and preliminary test a wearable, low-cost device enabling the objective monitoring of physical activity during sports training and patient rehabilitation. We have focused the design to monitor *f*_R_, HR, and movement cadence during running because it is a very common activity in several sports and rehabilitative programs and it requires particular attention in the sensor's selection and placement, more than in other sports activities.

## 2. Materials and Methods

The proposed device is composed of a hardware module, which allows the synchronous acquisition of *f*_R_, HR, and motion cadence, and a software module, in charge to manage the communication and data transfer.

### 2.1. Hardware Module: Sensor Selection

In the following lines, we will carefully review the technological solutions available on the shelf to measure *f*_R_, HR, and motion cadence. Each solution will be discussed to identify the one better fitting with the aim of this work.

#### 2.1.1. Respiratory Frequency Monitoring

The *f*_R_ may be estimated indirectly, by measuring parameters physically correlated with the breathing activity, such as changes in thorax circumference, thorax cross section, or transthoracic impedance [11], or using the so-called airflow-based methods [12, 13], based on variables derived from the airflow produced during breathing. The indirect methods use several technological solutions like microphones (measuring the sound created by turbulence that occurs during respiration) [14] or using straps embedding several sensors to measure the thorax expansion [15–17]. These solutions are quite tricky to be used during training due to the artifacts caused by movements and the environmental noise. The airflow-based methods measure differences between inspired and exhaled air; indeed, exhaled air is warmer, has higher humidity, and contains more CO_2_ than inspired air does. These parameters are usually monitored using temperature, hygrometers, and infrared absorption sensors [18]. Several commercially available solutions use a mask over the mouth and nose to convey the airflow or a thermocouple under the nose, measuring the change in temperature of the air as it is inhaled and exhaled [19]. These solutions, despite being quite accurate, cannot be adopted in physical training monitoring. A more straightforward direct way to assess only *f*_R_ consists of measuring the flow expired by the subject by using an accurate flowmeter for low-rate flows positioned over the mouth [12, 20]. This solution allows the easy detection of exhalation if the breathing is properly conveyed to the sensor, but it cannot be used to monitor the inspiration phase. Indeed, to convey the air toward the sensor during inspiration, it is necessary to use a face mask, which can be obtrusive for patients and athletes during physical activity.

Since we are interested in monitoring only the frequency of respiration, the use of low-rate flowmeters with a conveyor for exhaled air appears to be the most suitable choice due to its accuracy, easiness of use, and overall level of comfort.

#### 2.1.2. Heart Rate Monitoring

HR can be monitored using different techniques based on the electrocardiogram (ECG), which is primarily used in clinical applications since it is strongly affected by a baseline drift, which may be ascribed to a nonoptimal contact of the strip with the body and motion artifacts [21, 22].

**Figure 1:**
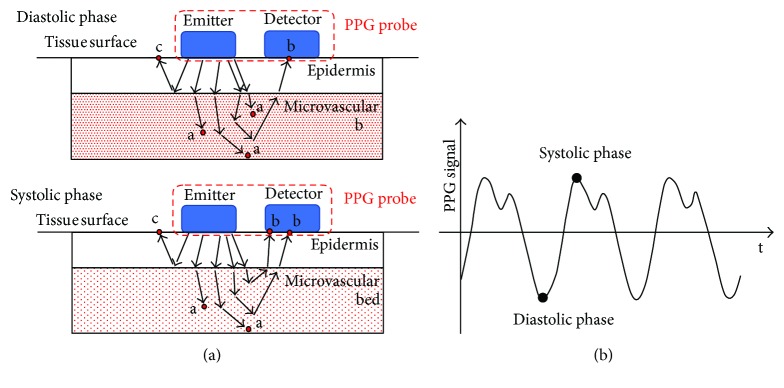
(a) Example of a PPG reflective probe. The couple emitter-detector is placed in direct contact with the tissue surface to reduce the optical noise. The amount of light absorbed by the tissue (red dots marked with a) depends on blood volume changes in the microvascular bed of the tissue; it is lower for small volumes (systolic phase) and higher for significant volumes (diastolic phase). (b) Typical PPG signal.

Moreover, even if the ECG method is accurate in clinical settings and static conditions, it is not very appropriate for HR monitoring during fitness and rehabilitation due to its complexity and cost. A promising, low-cost technique is represented by photoplethysmography (PPG), which consists in the shining of selected regions of the body (typically earlobes, fingers, wrist, or arm) and in the measurement of the perfusion of blood in the dermis and subcutaneous tissue by capturing the different amounts of refracted light [23–25]. A matched pair of emitter-detector is typically used: they can be placed in opposite positions (transillumination configuration) or adjacently (reflective configuration) as shown in Figure 1.

Photons (red circles in Figure 1) may be absorbed by tissues (a), scattered (c), or reflected to the detector (b). The absorption strongly depends on the amount of blood in the illuminated region, which in turn depends on the cardiac phase: the more blood there is in the illuminated region (diastolic phase), the more light is absorbed by hemoglobin, and the less light is reflected [26].  Figure 1(b) reports a typical PPG signal. The black circles correspond to the two main phases of the cardiac cycle: the systolic phase when the contraction of the blood vessel wall moves blood ahead, reducing the local volume of blood, and the diastolic phase when the blood vessel wall is relaxed and the volume of blood in the capillary bed is maximum. The main limitation for the application of such sensors during physical activity is their sensitivity to patient/athlete movements and/or probe-tissue displacement [27].

Indeed, these movements can modify the amount of light detected, creating what is known as motion artifact (MA). To overcome this limitation, it is necessary to use a fastening system to keep the sensor firmly in contact with the skin, select a location of the body not directly involved in the movement performed during training, and use an appropriate technique for MA reduction.

#### 2.1.3. Motion Cadence Monitoring

**Figure 2:**
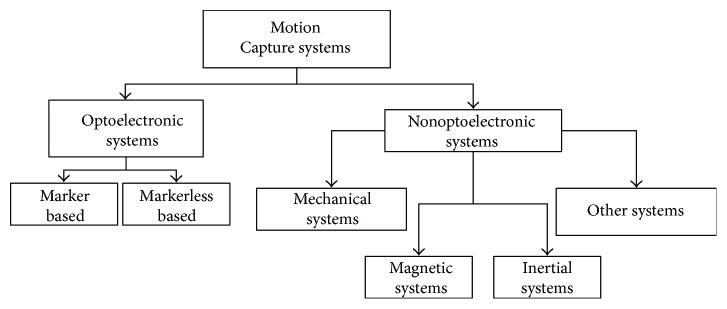
Systems for motion capture: a taxonomy.

There are several technological solutions and systems to monitor human movement and cadence. They operate on entirely different physical principles, with different performance characteristics. As shown in [28], there is not a single technology that can fit all needs, but each application defines the best one to be implemented.  Figure 2 reports a taxonomy of the main systems used for motion analysis that can be grouped into two main classes: optoelectronic and nonoptoelectronic systems (see [29, 30] for a detailed review).

Marker-based optoelectronic systems use high-frequency cameras and are generally considered complex and expensive and not suited to be used in an unstructured environment [31, 32]. Even if markerless systems are cheaper than marker-based solutions, they are sensitive to lighting conditions and require the subject to be always captured by the camera. However, nonoptoelectronic systems that group different physical principles can be used to monitor movement. Among others, mechanical sensing techniques are quite bulky and can limit the mobility of the subject. Similarly, magnetic sensors are not suited for physical activity monitoring because their range of measurement is small and the sensors require cables, thus limiting the movement of the subjects [33, 34]. Kinematic variables may also be measured by the mean of inertial systems [35]. In particular, microelectromechanical accelerometers allow to estimate the acceleration of movements measuring the relative displacement of a small mass (few *μ*g) with respect to the fixed external case and, merging information coming from accelerometers, gyroscopes, and magnetometers (the so-called inertial-magnetic unit or IMU sensors), they allow to reconstruct the orientation of the sensor in the space with a good level of accuracy [36]. Several configurations with different levels of complexity are possible, depending on the aim of the study. The reconstruction of the body kinematics requires the use of a network of sensors, worn by the subject in predefined positions and calibrated using a specific procedure, as well as biomechanical models to estimate the kinematics of articular joints [37]. The extraction of elementary information like step cadence can be performed by using a single accelerometer. The adoption of one sensor is good for prolonged measurements and monitoring because it has a small size (few millimeters) and it weighs a few grams; moreover, it can be easily worn and, thanks to its moderate electrical consumption, it can be supplied using relatively light and small batteries for a long period. Among the other systems, there are devices based on a combination of the previous techniques. For example, the Wii Remote is a commercial system which uses inertial and optical sensors to measure human motion [30]. These systems can be used in real-time application but require subjects to remain close to the optical system, thus reducing their use for motion monitoring during real exercise conditions.

#### 2.1.4. Selected Solutions

The analysis of the main techniques used to date to measure *f*_R_, HR, and motion cadence has allowed identifying the main pros and cons of each technology. Keeping in mind the specific application of our system and the main constraints it needs to fulfill to be effectively used for physical activity monitoring, three promising technologies have been selected among those presented. We decided to develop a sensor module instrumented with a PPG sensor to measure HR, an orifice flowmeter connected to a differential pressure sensor via two static taps to measure the pressure drop correlated with the exhalation/inhalation and then the *f*_R_, and a single triaxial accelerometer to both measure step cadence and reduce MAs from the PPG signal.

The automatic detection of MAs and its separation from pulse recordings is a nontrivial exercise in computer signal processing mainly due to their significant band overlapping. Numbers of solutions have been proposed ranging from moving average filters [38–40] to adaptive algorithms (least mean squares adaptive algorithm [41–45], Kalman filters [46], time-frequency methods and wavelet transform [47, 48], principal component analysis [49]); the main pros and cons are detailed in [50]. Within others, the software used to analyze the HR from the proposed device uses the normalized least mean squares (NLMS) adaptive algorithm presented in [41]. Such kind of filter has high computational efficiency, making it the perfect candidate for real-time applications by using the accelerometer data as reference signal.

**Figure 3:**
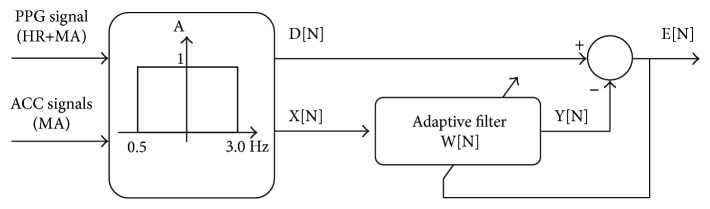
Block diagram of the NLMS adaptive filter used. The coefficients *W*[*n*] of the filter are tuned according to the equations in the body of the text.

Figure 3 reports the block diagram of the implemented algorithm. Both accelerometer and PPG signals are bandpass filtered between 0.5 and 3 Hz, that is, the band of the HR signal [42]. The acceleration signal is digitally filtered and subtracted to the PPG-filtered signal (D[N]) to remove MAs and obtain the E[N], that is, the reconstructed HR signal. The digital filter coefficients are adaptively tuned according to

(1) $$\begin{array}{c} W\left[n+1\right]=W\left[n\right]+\mu \left[n\right]x\left[n\right]e\left[n\right]. \end{array}$$

*μ*[*n*] represents the step size of the filter and is calculated as in

(2) $$\begin{array}{c} \mu \left[n\right]=\frac{b}{a+x^{T}x}, \end{array}$$

where *b* and *a* are two constant coefficients; see [51] for details.

### 2.2. Communication and Data Transfer Protocol

To allow the real-time objective measurement of relevant variables during physical activity, a communication protocol should satisfy the following main requirements:

- It must be a wireless solution (as in many cases, the sensors will be in different areas).
- It should allow connecting a small set of sensors to obtain synchronous data from them.
- It should provide low-power consumption.
- It should be based on standard hardware and protocols as much as possible.
- It should provide a sufficient throughput rate to guarantee a suitable sample frequency (at least 100 Hz).

To select the best solution for our application, we compared some of the most well-known standard suites for wireless personal area networks (PAN) and sensor networks in general. In Table 1, we compared the main technologies available, in detail: Bluetooth and its more recent low-consumption variant, Bluetooth Low Energy (BLE), Wi-Fi, 6LoWPAN/Thread, NFC, ZigBee, and nRF24 (by Nordic Semiconductor).

Bluetooth is one of the most known and spread M2M communication systems; BLE is a variant of the main Bluetooth specification, also known as Bluetooth Smart. It was created to provide lower consumption while maintaining a similar communication range. Wi-Fi is the most used technology for wireless connections with Internet access. It is based on the IEEE 802.11 standard, which allows a broader range of connectivity, allowing for wider networks. 6LoWPAN is a technology that aims to use IPv6 over the Internet of Things (IoT) and sensor networks, through the IEEE 802.11.4 standard. It does not define an application layer protocol, except for efforts such as the recently released Thread (backed by more than 50 companies). Near-field communication (NFC) is a set of protocols that allows the communication of two devices located in close range (a few centimeters). Currently, it is widely used for payment process or data exchange between smartphones. ZigBee is an alternative for 6LoWPAN which implements the full stack of protocols including the application layer. It is also based on the IEEE 802.11.4 standard. The idea behind ZigBee was to provide a simpler and less expensive alternative to Bluetooth and Wi-Fi. There are other solutions for the creation of small wireless networks, not necessarily based on a full standard stack of protocols. For instance, Nordic Semiconductor offers products that allow the creation of radio frequency-based communications using the same frequency as Wi-Fi in their nRF24 devices.

The comparison, presented in Table 1, suggests Bluetooth as the best technology to guarantee real-time application with a good spatial range and low consumption. Indeed, Wi-Fi-related technologies, even if highly spread in the market, require high power and need to structure the environment with access point to be used efficiently. Other solutions such as 6LoWPAN or ZigBee offer lower data rates, thus making real-time applications not possible. NFC technologies can be used in a tiny spatial range, and nRF24 technologies are not based on any standard and present a low market adoption for this kind of devices. Between Bluetooth and BLE, we have finally selected the first one, as, in this case, a higher data rate it is more important than a lower consumption.

Moreover, it has been demonstrated that the theoretical data rate of BLE is strongly dependent on the distance and it decreases very much also for small distances, well below the theoretical limit of 50 m [52, 53].

### 2.3. System Development

**Figure 4:**
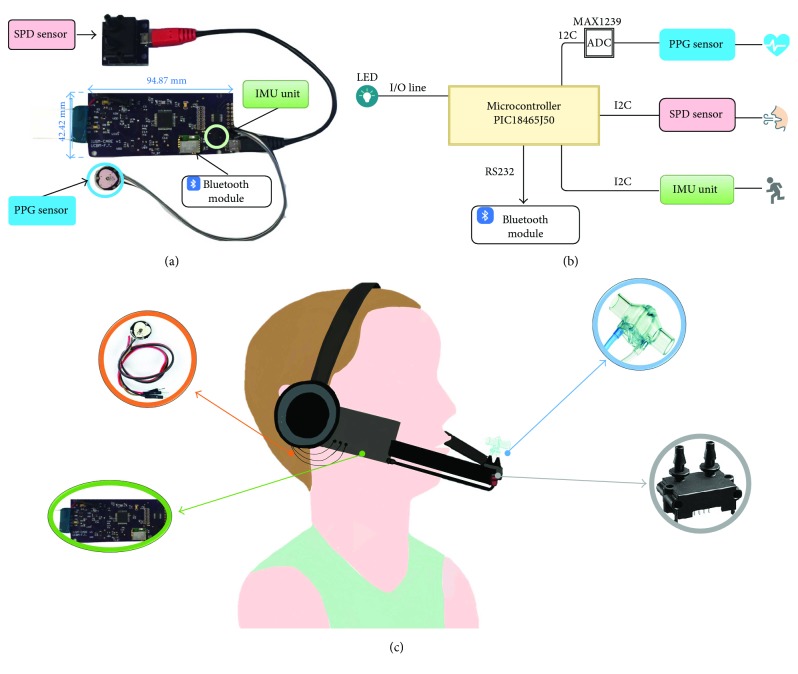
(a) Detailed view of the hardware module. (b) A functional diagram of the PIC-based board. The control unit acquires and collects data from the PPG sensor (through an analog-to-digital converter), the SDP sensor for respiratory rate, and the IMU unit for body movement. Data are sent to PC by the Bluetooth module. (c) Overall system sketch. The hardware module has been embedded into a headphone. The flowmeter is positioned 10 cm ahead the mouth, and its static taps are connected to the SDP sensor. The PPG sensor is positioned on the right earlobe with an ear clip. The IMU is integrated into the hardware module in proximity with the PPG sensor.

In the development of the proposed system, we carefully considered some designing constraints in the hardware part. Particular attention was paid to keep the weight and the size factor of the system as small as possible and to select the electronics to be compatible with low-voltage power supply reducing as much as possible the overall power consumption of the system, as suggested by [52]. The board (size: ~95 mm × 42 mm × 1.5 mm), shown in Figure 4(a), has been equipped with a local control unit (a microcontroller PIC18F46J50, by Microchip Technology Inc., current consumption 23.2 mA (settings: FOSC = 48 MHz, PRI_RUN mode, EC oscillator) and an inertial module (LSM9DS0 by STMicroelectronics, range up to ±58.86 m/s^2^, resolution 16 bit, maximum current consumption about 10 mA) for motion monitoring. It is also provided with two standard sockets to easily plug/unplug a PPG sensor (PulseSensor, World Famous Electronics llc, maximum current consumption 4 mA) and a commercial flowmeter (SpiroQuant P by EnviteC, Honeywell) connected to a differential digital pressure sensor (hereinafter named SDP sensor, model SDP610, pressure range up to ±125 Pa, accuracy < 0.04% full-scale, Digital I^2^C Output, maximum current consumption 6 mA).

Both accelerometer and pressure sensors are provided with a digital I2C interface while the signal of the PPG sensor is locally conditioned and analogically sent to a 12-bit ADC on the board (MAX1239, by Maxim Inc., maximum current consumption 230 *μ*A) connected with the local control unit via an I2C interface. The communication is locally timed by the control unit that is programmed to acquire data from sensors at 100 Hz. The acquired data are sent to a remote unit thanks to a Bluetooth V3.0 module (i.e., by the SPBT2632C2A module by STMicroelectronics, maximum current consumption 27.5 mA, in slave mode during data transmission at maximum throughput). All the electronics are supplied by a LiPo 3.6 V, rechargeable battery provided with an on-board charger. According to the maximum current consumption of the main components of the board, the electronic requires about 70 mA during data transmission. For this reason, we selected the LiPo battery GM302547_PCB, produced by PowerStream Corporation. This battery has a capacity of 320 mAh that guarantees more than 4 hours of continuous operation, that is, a time widely sufficient for a typical training session or rehabilitative procedure. A functional diagram of the electronic is presented in Figure 4(b).

The board has been integrated into a headphone provided with a microphone stick (Figure 4(c)). The microphone has been replaced with a commercial flowmeter that causes a pressure drop when the exhaled air flows through the restriction created by its orifice. The SDP sensor captures the pressure drop. The PPG sensor has been connected to the right earlobe, which is closer to the accelerometer sensor, thus allowing an easier compensation of MA.

**Figure 5:**
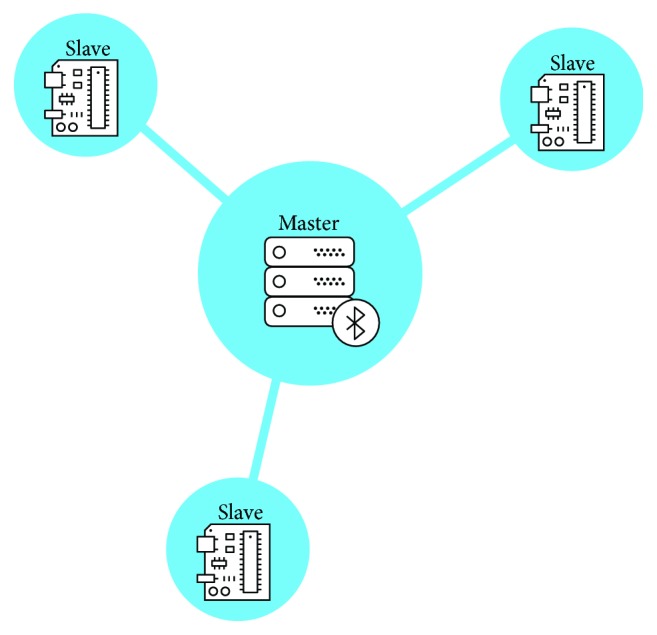
Piconet topology implemented through Bluetooth connection.

The Bluetooth connection has been used not only to guarantee M2M connection but also to create a local sensor network and test the possibility to use these sensors in a local network configuration, to promote social interaction which can foster the motivation to perform physical activities. Typically, Bluetooth connections are defined as point-to-point connections or as a piconet (a Bluetooth network where one element acts as a master, and there are a set of slave devices connected to it using a star topology). This last scenario is the one we tried to implement. As shown in Figure 5, the scenario is composed of a set of modules for physical activity monitoring (slaves) provided with a Bluetooth interface that send their data to a central device, the master of the network.

By following the classic client-server architecture [54], we also defined the master device as a server, to which all the slaves will connect, acting as clients. Consequently, our communication system is composed of clients, servers, and the interactions between them.

We used the radio frequency communication Bluetooth protocol (RFCOMM) and the Serial Port Profile (SPP) to establish connections between the master and each slave. Moreover, to standardize the interactions between clients and server, we defined a simple message set. It is composed of only six different types of message. Each one consists of a set of bytes representing different control fields to be shared between clients and server. All of them start with a byte representing the type of the message and end with a special ending byte value. The messages are as follows:

1. Initialization (Init) message: the first message sent from the client after the connection. It is used to advertise the information of the client—specifically, the number of sensors, the size of data each one can provide, and the frequency of data acquisition.
2. Configuration (Conf) message: after the Init message, the server sends a configuration message to each client. It consists of a list of sensors to be used in a specific data acquisition process.
3. Start message: a simple signal that notifies a client that should start the data acquisition and transmission to the server.
4. Stop message: as the start message, it is a simple signal to notify a client that it should stop acquiring and sending data.
5. Data message: this is each message containing sensor data, sent from each client to the server.
6. Sync message: it is a specific message defined to maintain the synchronization between the clocks of all the clients connected to the server at some point. These messages are sent periodically from the server, regardless of the sequence of data acquisition.

**Figure 6:**
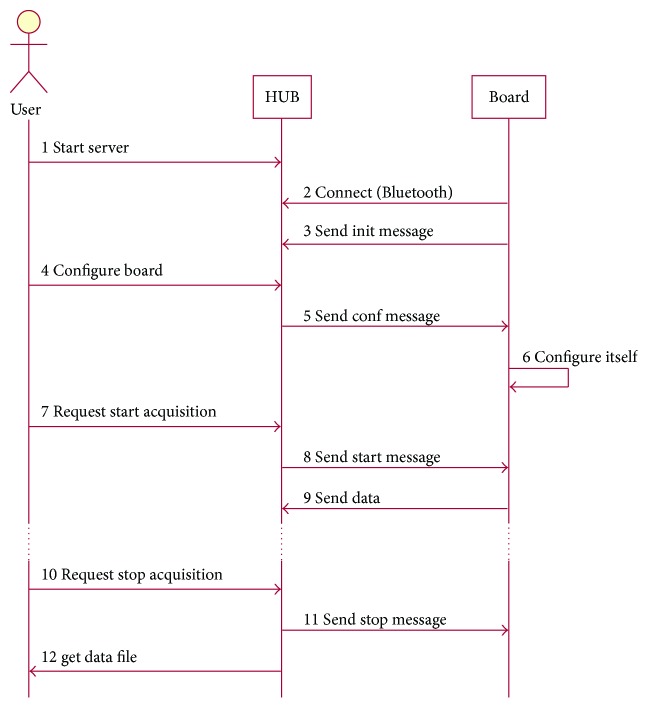
Sequence diagram for the application protocol.

Figure 6 represents the usual sequence of message exchange. The sequence starts right after the connection established to the server which is advertising a Bluetooth SPP service. In the diagram, HUB represents the server and master and Board represents each client and slave.

**Figure 7:**
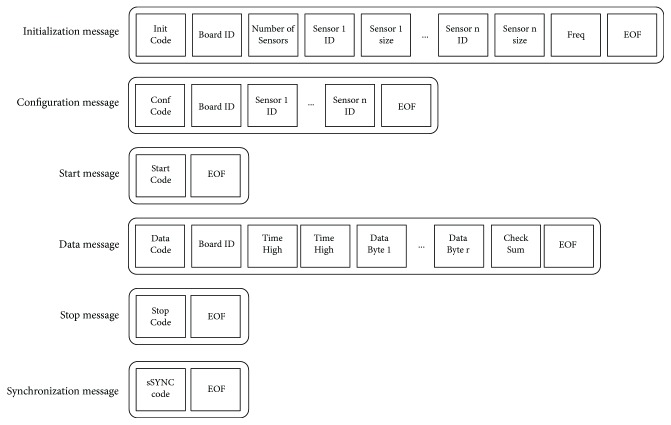
Message formats (bytes).

Figure 7 shows the different fields of each message. All fields are of 1-byte length, except for the Time (which is divided into 2 bytes) and the sensor data (which will depend on the data received from specific sensors, and therefore, it will be variable) in data messages.

**Figure 8:**
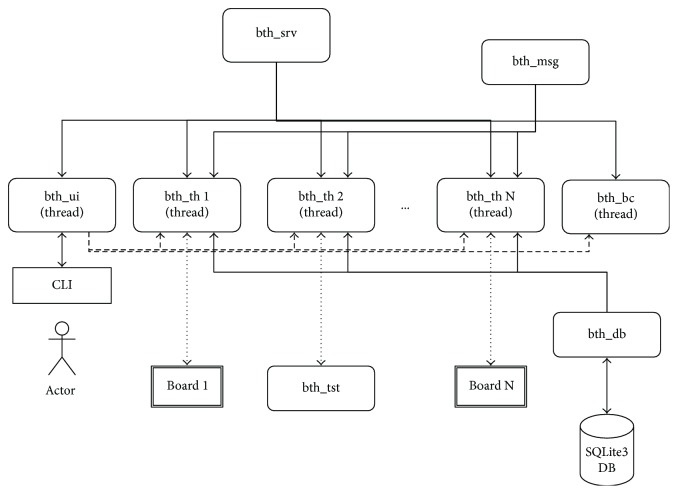
Module diagram of the prototype server implementation.

To prototype the server, we have used Raspberry Pi 3 Model B, which includes a Bluetooth adapter and offers a simple and easy way for programming. The designed server could be easily ported to a smartphone or any other Bluetooth-capable device. We developed the server over the Raspbian operating system, using the PyBluez library and the Python language. The server is composed of a series of threads (bth_th), which manage each point-to-point connection and defines the messages of the protocol in a dedicated module (bth_msg). It also offers a command line interface (bth_ui) for the user interaction and can register and store board configurations using a simple SQLite database.  Figure 8 shows the module diagram of the implemented server.

There are several possible solutions to guarantee time synchronization among different devices connected to the server [55–57]. Some of them present the inconvenience of needing dedicated hardware (i.e., GPS or Internet-connected adapters to use the Network Time Protocol (NTP)), while others rely on local protocol implementations (i.e., Clock Synchronization Protocol (CSP), an optional feature of the Bluetooth Health Device Profile (HDP) [58]). In our case, CSP is not supported and it is also not possible to use dedicated hardware, so we have tried to implement a simple protocol based on sending broadcast messages from the server to the clients to reset the client clocks periodically. As RFCOMM/SPP uses point-to-point connections, it is not possible to use “real” broadcast messages (i.e., just one message sent to the shared communications medium and read by all the devices listening). Instead of that, we send a copy of the synchronization message to each client sequentially. If the process is fast enough (i.e., if it lasts less than the sampling interval, 0.01 s), it will allow keeping the client clocks synchronized during all acquisition.

## 3. Results and Discussion

The device developed for monitoring respiratory frequency, heart rate, and step cadence presented in the previous section can be used as a stand-alone system, connected to a smartphone, or as a node of a local network provided with a central master.

We have tested the ability of the device to be efficiently used as a wearable monitoring device in resting condition and during physical exercises (i.e., running on site) and, subsequently, verified the possibilities to implement a network configuration using a simple Bluetooth V3.0 adapter. In particular, we tested the possibility to obtain synchronous data from the nodes of the network.

To verify the reliability of the system and its suitability to monitor physiological parameters during physical exercises, two preliminary data collections have been carried out: (i) in the laboratory (by simulating the breathing pattern) and (ii) by collecting data from one healthy volunteer. Lastly, we tested the synchronization among different devices using both a synchronization message serially sent to each device and a dedicated broadcast thread.

### 3.1. Simulated Breathing Pattern

To test the ability of the proposed device to detect the pressure drops correlated with the breathing and then to calculate *f*_R_, two breathing patterns were simulated by using a mechanical ventilator (Servo Ventilator 900C, Siemens-Elema AB, Sweden). The ventilator was used in the continuous mandatory ventilation (CMV) mode, in which breaths are delivered based on set variables (e.g., *f*_R_, tidal volume, pauses between phases) [59].

The flowmeter connected to the SDP sensor was placed downward the end of the Y-piece of the breathing circuit that delivers the air generated by the ventilator, at the same distance the mouth is when the flowmeter is mounted on the headphone (~10 cm).

**Figure 9:**
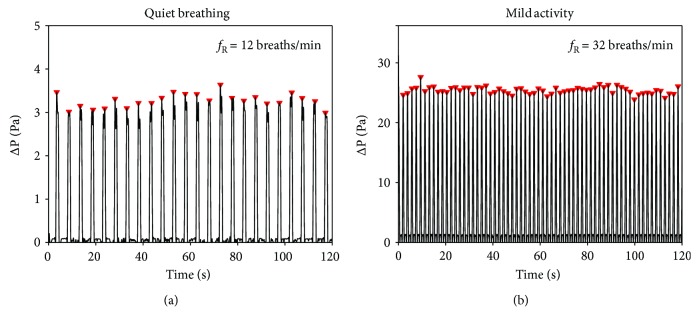
Pressure drop recorded by the SDP sensor integrated into the proposed device at two different breathing patterns delivered by a mechanical ventilator simulating a quiet breathing (set *f*_R_ = 12 breaths/min) and the breathing during physical activity (set *f*_R_ = 32 breaths/min).

Two different breathing patterns were generated with the ventilator: (i) quiet breathing, by delivering air for 120 s with a set *f*_R_ of 12 breaths/min, tidal volume of 770 mL (minute volume of 9.2 L/min), and 25% of inspiratory pause and 10% of pause between inspiration and expiration phases, and (ii) mild activity, by delivering air for 120 s with a set *f*_R_ of 32 breaths/min, tidal volume of 800 mL (minute volume of 25.9 L/min), and 25% of inspiratory pause and with a 10% of pause between inspiration and expiration phases. Breathing was real-time measured by the proposed device. Pressure drops collected by the device are shown in Figure 9, together with the maximum points automatically detected by the ad hoc developed algorithm used to calculate *f*_R_. The device detected 24 breaths during quiet breathing (12 breaths/min) and 64 breaths during physical activity (32 breaths/min). The pressure drop recorded during quiet breathing was one order of degree lower than the drops recorded during the activity (up to ~3 Pa versus up to ~30 Pa).

### 3.2. Data Collected on the Healthy Volunteer

One healthy male (age = 36, weight = 76 kg) was asked to wear the proposed device on the head, to place the flowmeter ahead of the mouth (at a distance of ~10 cm), and the PPG sensor was connected to the right earlobe.

**Figure 10:**
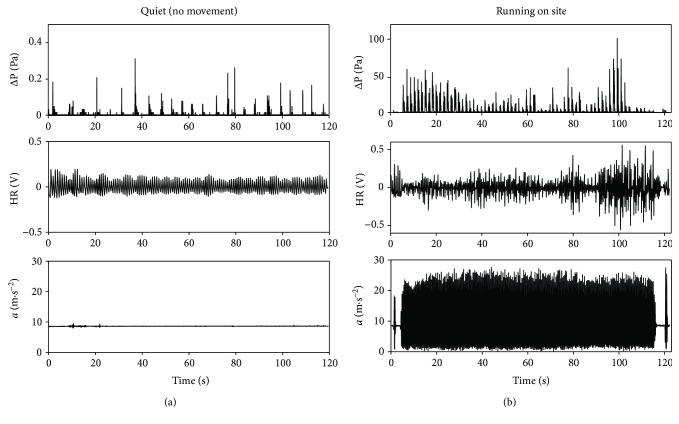
Data collected during the preliminary test on the healthy volunteer (i.e., pressure drops, accelerations, and PPG signals). The proposed device detects movements, HR, and breathing, both during quiet breathing (no movement) (a) and the running phase (b).

The test consisted of two trials lasting 120 seconds each one. In trial 1 (quiet breathing, without movement), the subject was asked to be relaxed and to breathe with the mouth. The breathing, heart rate, and movement of the subject were collected by the device to define a baseline. Then, in trial 2 (running on site), the subject was asked to run on site with a self-paced cadence. Breathing, HR, and movements were real-time collected with a laptop connected via Bluetooth to the proposed device. The laptop was about 3 m distant from the device. In the typical application scenario, the user's smartphone will replace the laptop and the distance device-smartphone will be reduced at about 40–50 cm. Despite such overestimation in the distance, we did not observe any data loss.  Figure 10 shows data collected by the proposed device during the two trials.

**Figure 11:**
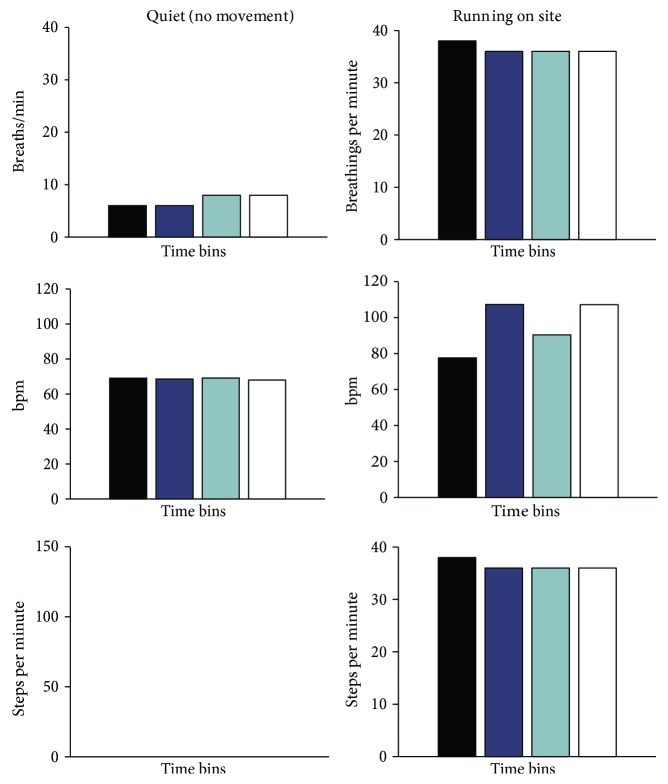
Data collected from the healthy volunteer during the quiet breathing (no movement) and running trials with the proposed device. The numbers of breaths, beats, and steps per minute are presented in 4 time bins lasting 30 s each one.

To measure the performance of the device to track the subject's motor behavior activity during the exercise, the breaths per minute, beats per minute, and steps per minute values were divided into 4 time bins lasting 30 seconds (see Figure 11).

#### 3.2.1. Quiet Breathing Trial

During quiet breathing, *f*_R_ was calculated by the pressure drop signal (Figure 10(a)). All the maxima were detected, and then all the time intervals (*T*_interval_^*i*^) between two consecutive maximum points were calculated. So, the instantaneous *f*_R_^*i*^ is calculated as in

(3) $$\begin{array}{c} {f_{R}}^{i}=\frac{60}{{T_{interval}}^{i}}. \end{array}$$

The subject's *f*_R_ was 12 ± 1 breaths/min on average.

The HR has been estimated in each 30 s lasting time bin according to

(4) $$\begin{array}{c} HR=\frac{number\text{ of }peaks}{30}\cdot 60. \end{array}$$

Peaks were identified from the raw signal and from the signal filtered with the adaptive filter discussed above (see (2) and (3)). We obtained no differences in the 4 time bins because the adaptive filter removes motion artifacts, which are not present in this case.

The volunteer showed an average value of 69 ± 1 bpm with a small standard deviation during the 120 s of data collection (1 bpm). Moreover, no steps were detected by the device during this trial (the accelerometer signal is always ~9.81 m·s^−2^, as shown in Figure 10), as expected.

#### 3.2.2. Running On-Site Trial

During this trial, we tested the ability of the system to measure steps and breathing frequency reliably. Compared to values gathered without movements, when the subject starts to run, values increased significantly. During the running trial, the subject was recorded with a full HD camera embedded in Huawei Honor 6 Plus (Android 4.4.2) at 30 frames per second (fps). The subject was asked to jump before starting to run, and just after he had ended, respectively, at second 2 and at second 120 (see Figure 10). This procedure allowed to identify data corresponding to the running session clearly and to synchronize data with video. In detail, we realigned the frame at which foot contacted the floor after the first jump with the first peak in the acceleration trace. The subject's average *f*_R_ was higher than that in the quiet condition, with an average value of 36.5 ± 1 breaths/min (minimum value: 36 breaths, the last three time bins; maximum value: 38 breaths, the first time bin). His HR increased too, with an average value of 96 ± 14 bpm (minimum: 78 bpm in the first time bin; maximum: 107 bpm in the second and last time bins). The efficacy of the adaptive filter can be fully appreciated in this condition, that is, when motion artifacts are more likely.

**Figure 12:**
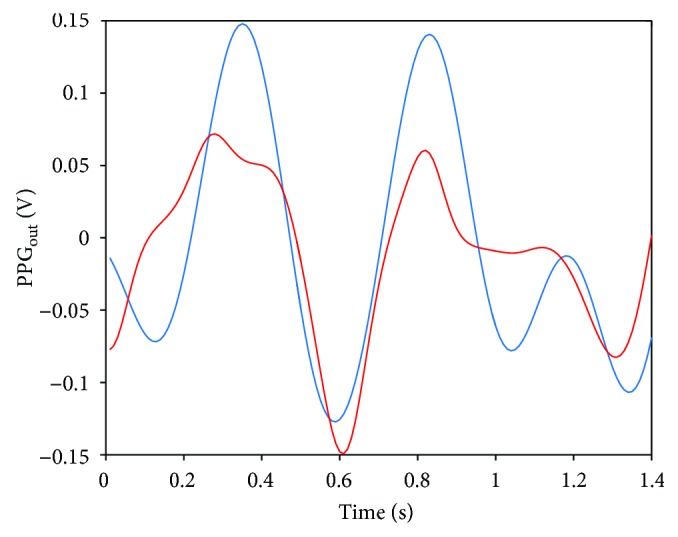
PPG output: the blue line is the raw signal filtered between 0.5 and 3.0 Hz; the red line is the PPG output after the NLMS filtering.

Figure 12 reports the effect of the filter on the morphology of the PPG signal. The blue line is the PPG signal, filtered with a bandpass filter between 0.5 and 3.0 Hz; the red trace is the PPG signal filtered with the NLMS adaptive filter. As can be observed, the NLMS adaptive filter reduces signal distortion due to motion artifacts and produces in output a signal which resembles the theoretical one presented in Figure 1. A difference, even if not significant, can be also observed comparing the HR estimations (NLMS filtered versus bandpass filtered). In the four time bins, we have, respectively, 79 ± 6 bpm versus 92 ± 8 bpm, 108 ± 8 bpm versus 102 ± 7 bpm, 90 ± 5 bpm versus 93 ± 5, and 107 ± 9 bpm versus 114 ± 7 bpm.

The recorded video was analyzed with a free video analysis and modeling tool (Tracker v4.92) [60]: the contacts between the shoe of the volunteer and the floor (number of steps) were manually identified by a frame-by-frame analysis. The number of the steps identified by this analysis was compared to the number of steps calculated from the signal recorded by the accelerometer integrated into the proposed device. Both systems detected 270 steps in total. All the contact instants (steps) were identified in both signals; then, all the time intervals (*T*_interval_^*i*^) between two consecutive steps were calculated. The mean absolute error (MAE) was used to measure the differences between all the time intervals calculated by the proposed device and by the video as in

(5) $$\begin{array}{c} MAE=\frac{\sum_{i}^{n}\left|{{T_{interval}}^{i}}_{device}-{{T_{interval}}^{i}}_{video}\right|}{n}. \end{array}$$

The MAE was 0.042 ± 0.029 s (expressed as mean ± standard deviation). During the whole trial, the accelerometer detected an average time interval between steps of 0.407 ± 0.048 s whereas the event detected by the video showed an average time interval of 0.412 ± 0.047 s. The difference between the standard deviations demonstrated the good performance of the proposed system in tracking variations of the cadence over time. The proposed device evidenced an average activity pace of 147 ± 3 steps per minute.

### 3.3. Synchronization Tests

We tested the synchronization method described in the previous section. To test the first implementation, we measured the time necessary to send a message to various connected boards from our server. We implemented a synchronization response message (used only for testing purposes) which was set by the clients immediately after receiving the sync message. In the first tests, we sent 300 sync messages (one message each second) having 2 active connections, waited for the responses, and measured the difference of time between the reception timestamps (measured in the server) for each one of the clients. If the difference between timestamps was low enough and stable through time, this would indicate that the adopted implementation was feasible for synchronization.

**Figure 13:**
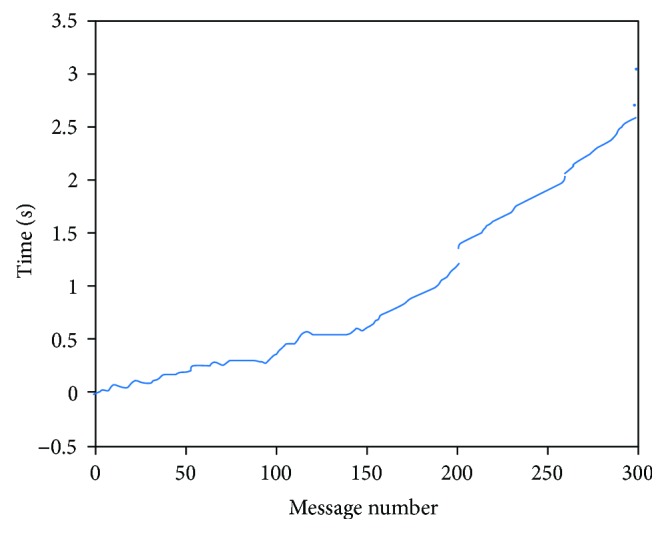
Difference of times measured on synchronization response reception from two different boards using independent threads in the server.

The approach proposed for synchronization gets a cumulative error between the reception times of the response from each board (Figure 13). This is probably derived from the independence of execution between threads, which makes the sending of each message virtually asynchronous.

**Figure 14:**
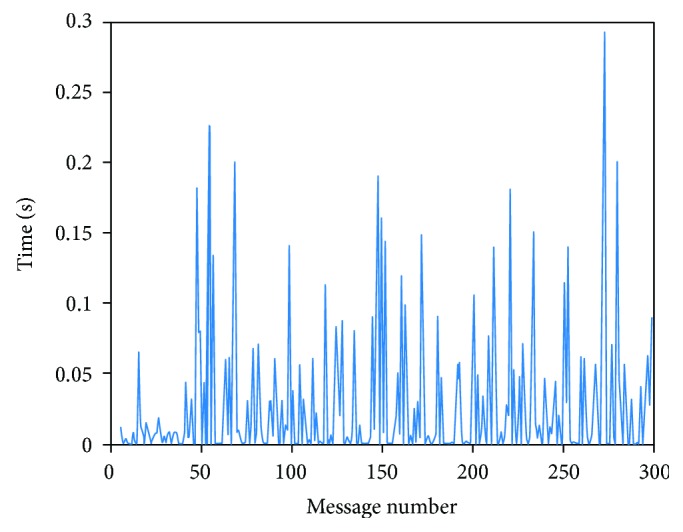
Difference of times measured on synchronization response reception from two different boards using a dedicated thread in the server.

To avoid this error, we developed the broadcast thread (bc_bth) shown in Figure 14. This thread allows us to send all the synchronization messages to each board using a loop, fixing the time interval from each sending. Repeating the last test, we obtained the results shown in Figure 14.

**Figure 15:**
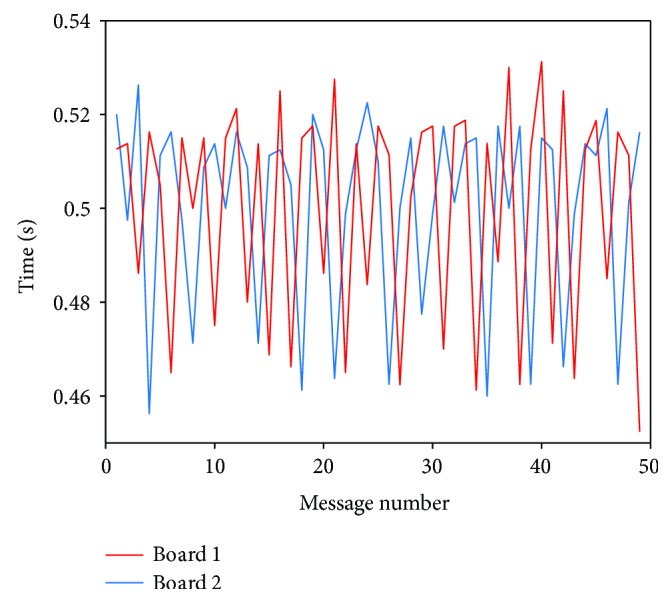
Difference of times measured on the server from synchronization messages sent by 2 different boards.

In this case, there is no drift, and therefore, the differences are independent for each pair of messages. Notwithstanding the absence of drift, the results show that there is a significant variability in the time delay and, more importantly, this figure shows how these delays are higher than the sampling frequency. In the second implementation, we proposed the use of synchronization messages sent from the clients at the beginning of the communication to calculate an offset in the server for each board, according to its clock. We tested this implementation by sending 50 synchronization messages each 0.5 second from 2 different boards and measuring reception times from the server.  Figure 15 shows the difference between each two received messages for each board.

The results show that, although all messages are received in about 0.5 seconds (average values are 500.46 ms and 499.85 ms for boards 1 and 2, resp.), there is a high variability between each interval of time (the standard deviation is 20.69 and 23.13, resp., a difference of almost 80 ms between the minimum and maximum measured values: 70 ms for board 1 and 78.8 ms for board 2).

If the measurements are correct, the difference in delays prevents us from using this information to find a reliable offset value related to the server clock. Since the high variability in these measurements is being obtained from the application layer and therefore could depend on the operative system of the server, we repeated these tests using sniffers (specifically wireshark and hcidump) which are designed to capture messages in the Host Controller Interface (HCI) (i.e., in the interface between the host system and the Bluetooth controller).

Although the times were slightly lower than the ones measured at the application layer, the variability persisted. More tests should be performed to determine where the cause of the variation between messages is. As it can be a result of low-level tasks in the Bluetooth adapter (in both clients and server), it would be a right approach to measure times as close as possible to the hardware, as it has been done in [61].

## 4. Conclusions

In this paper, we designed and preliminarily evaluated a wearable system for continuous real-time monitoring of physical activity.

The proposed system has been designed, built, and tested, and thus, the description of each module and the adopted methodological choices have been reported. Hardware and software modules compose the system. A variable-orifice meter combined with a differential pressure sensor (SDP sensor) is used to collect the pressure drop and to estimate *f*_R_; a commercial photoplethysmographic sensor (PPG) is used to collect the heart rate; an IMU unit is used to monitor the body movement and then to calculate the steps during walking or other activity. A PIC-based board was designed, made, and tested to acquire and collect data from the three hardware units and to transmit data via a Bluetooth module. The size of the board (~95 mm × 42 mm × 1.5 mm) allows the integration of hardware modules on a headphone which is comfortable to wear during activity. Bluetooth allows establishing real-time communications between the proposed system and a centralized device such as the user's smartphone. We designed and developed a Bluetooth-based communication scheme following a client-server model to verify the possibility of simultaneous real-time acquisition from multiple wearable systems.

To test the system for physiological parameter monitoring, two preliminary data collections have been carried out: (i) in the laboratory (by simulating the breathing pattern with a mechanical ventilator) and (ii) by collecting data from one healthy volunteer.

The experimental results show the feasibility of the proposed device to identify the exhalation phase of the breathing continuously and to extract the *f*_R_ value from the pressure drop collected by the board. All the breaths simulated with the mechanical ventilator were correctly real-time detected both in quiet breathing conditions (set *f*_R_ = 12 breaths/min) and in physical activity conditions (set *f*_R_ = 32 breaths/min) by the proposed device. During the trials on the healthy volunteer, the device reported an average value of 12.1 breaths/min at rest and 36.5 breaths/min during the running trial. These values are within the physiological range [62]. Compared to other solutions adopting textile substrates or similar, which can be affected by movement artifacts due, for example, to slipping or movement during activity [11], the pressure drops collected by the proposed system (up to 50 Pa) allow the easy identification of pressure peaks and the extraction of *f*_R_ values. Moreover, differently from other direct methods adopting spirometers and face masks, the proposed solution does not cause discomfort for the user (no interferences with the freedom of movements) and does not disrupt the regular breathing pattern during measurement. Despite the absence of a reference instrument for the detection of HR, values collected by the proposed system are within the physiological range and increase during the exercise [63]. Data show that the proposed device can adequately monitor movements and step cadence: no movements were detected during quiet breathing, that is, when the subject is in resting state, while the same number of steps was recorded by the proposed device and the reference system during running activity. Further trials will be necessary in the future to evaluate the performance of the proposed device in collecting heart rate values by comparison with a reference instrument. The proposed device can be used as a stand-alone system, connected to a smartphone or as a node of a local network provided with a central master. Our preliminary findings raise some concerns about the possibility to use standard Bluetooth module available on smartphones to develop this second configuration. Indeed, it provides a RFCOMM/SPP point-to-point connection that does not allow a “real” broadcast messaging. For this reason, we investigated alternative possibilities, but the delay we obtained does not allow the use of these techniques for this kind of application. A possible solution to overcome this limitation is the use of a docking station at the beginning of the exercise session to simultaneously start the acquisition from the remote nodes. This solution may guarantee the synchronization among the different modules for a limited amount of time due to the clock drifts of each board. If effectively implemented, this second configuration could be used to simultaneously collect performance data from different athletes in both team and endurance sports, as well as in clinical and injury rehabilitation settings to monitor and motivate patients during their training program.

## Figures and Tables

**Table 1:** Wireless communication technology comparison.

	Bluetooth	BLE	Wi-Fi	6LoWPAN Thread	NFC	ZigBee	Other (nRF24)
Power consumption	Low (>30 mA)	Very low (<15 mA)	High (60 mW)	Low	Very low (<15 mA)	Medium (<1 *μ*W to 50 mW)	Very low (15 mA)
Max range	50 m/100 m	50 m	100 m	30 m	0.2 m	100 m	30 m
Standard	802.15.1 (originally)	802.15.1 (originally)	802.11	802.15.4	ISO/IEC 18092, ISO/IEC 14443-2,3,4	802.15.4	—
Market adoption	Very high	High	Very high	Low	High	High (in industrial developments)	Low
Max data rate	24 Mbps	1 Mpbs/2 Mbps	300 Mbps	250 Kbps	424 Kbps	250 Kbps	1 Mpbs/2 Mbps
